# Imaging and surgical predictive factors for postoperative hemorrhage after partial nephrectomy and clinical results of trans-arterial embolization

**DOI:** 10.1097/MD.0000000000023581

**Published:** 2021-01-22

**Authors:** Caipeng Qin, Xin Zhi, Fei Wang, Qing Li, Jian Gao, Shijun Liu, Tao Xu

**Affiliations:** a Department of Urology; b Department of Interventional Radiography, Peking University People's Hospital, the Second Clinical Medical College of Peking University, Beijing, China.

**Keywords:** hemorrhage, partial nephrectomy, predictive factors, renal function, trans-arterial embolization

## Abstract

Partial nephrectomy (PN) has been established as the standard treatment for T1 renal tumors, and postoperative hemorrhage due to vascular complications is a rare but potentially life-threatening complication reported after PN. Thus, this study evaluated the imaging and surgical factors associated with postoperative hemorrhage after PN and the clinical results of trans-arterial embolization. A retrospective review of the institutional PN database was performed from May 2012 to January 2019, revealing that we performed 810 PN procedures at our institution. In total, 12 patients were referred to the interventional radiology department for vascular complications after the procedure. Patients with and without transarterial embolization (TAE) were age- and sex-matched with 56 patients. Preoperative imaging characteristics and operative details were considered. Univariable and multivariable analyses were used to test their eventual association with the occurrence of hemorrhage. Furthermore, renal functions at diagnosis, after operation or embolization for TAE cases, and at the last follow-up were recorded. A diagnosis of hemorrhage was made at a median of 4 (range, 0–25) days after surgery. The majority of patients (50%) presented with gross hematuria. *T* test revealed higher renal tumor-parenchyma contact area (TPA) (*P* = .0407), Length-A (*P* = .0136), Length-P (*P* = .0267), operation time (*P* = .0214) and estimated blood loss (*P* = .0043) in patients with hemorrhage than in controls. Binary logistic regression analysis identified TPA (*P* = .048) and estimated blood loss (*P* = .042) as independent predictors for postoperative hemorrhage with an area under the ROC curve of 0.705 (64% sensitivity and 79% specificity). In conclusion, the occurrence of hemorrhage after PN was associated with a larger TPA and more estimated blood loss during the procedure. In patients who underwent selective TAE, renal function remained comparable with that of controls.

## 1 Introduction

Partial nephrectomy (PN) is a well-established treatment for renal masses,^[1,2]^ offering acceptable preservation of renal function without compromising oncological outcome.^[3–5]^ However, it has been widely reported that PN is associated with a greater risk for life-threatening postoperative hemorrhage than other treatments, mostly in the development of iatrogenic vascular lesions, such as symptomatic pseudoaneurysms, arteriovenous fistulas, or arterial bleedings.^[6,7]^ Therefore, there is an urgent need to identify clinical predictive factors of hemorrhage.

Trans-arterial embolization (TAE) is a minimally invasive and effective treatment option for vascular complications after PN.^[8,9]^ However, the effect of TAE on short- and long-term renal function remains equivocal. Potentially, TAE results in certain renal parenchymal volume loss, and contrast agents used during TAE may also impair renal function. Thus, the renal functional outcomes in patients undergoing TAE should be assessed.

## 2 Materials and methods

### 2.1 Patients

This study was approved by the Peking University People's Hospital Institutional Review Board. Informed consent was waived owing to the retrospective nature of the study. We retrospectively assessed consecutive patients undergoing PN at our institution between May 2012 and January 2019, and a total of 810 PN procedures were performed at our institution. Among them, 12 patients with postoperative hemorrhage and subsequent TAE were identified and matched with patients (n = 56) without this complication (matched by sex and age). The electronic medical records of all patients were carefully reviewed. Patient demographics and baseline characteristics (including age, gender, tumor volume, side, and histology) (Table 1), imaging features (including renal tumor-parenchyma contact area [TPA], Length-A and Length B), surgical variables (including operative time, estimated blood loss and warm ischemia time) and renal function data (preoperation, postoperation or TAE and at last follow-up) were collected and analyzed. For patients with TAE, clinical manifestations and details of angiographic findings were collected as well.

**Table 1:** Patient characteristics and clinical presentations.

Parameter	Controln = 56	Embolismn = 12	*P* value
Ag (yr)	58.8 ± 11.0	55.3 ± 12.4	.338
Sex (M and F)	48,8	10,2	1.000
Tumor size (cm) Median (range)	2.8 (1.0–6.0)	3.0 (0.8–8.7)	.272
Tumor side			
Right	31	3	.056
Left	25	9	
Histology			
Clear cell cancer	47	7	.110
Others	9	5	
Follow-up (mo) median (range)	16 (5–46)	12 (0–44)	

### 2.2 TPA, length-A, and length-P measuring methodology

All measurements were made manually on contrast-enhanced computerized tomography using a GE Advantage Workstation software (versions 4.6p, American.ge.aw) in the axial plane.

**Figure 1:**
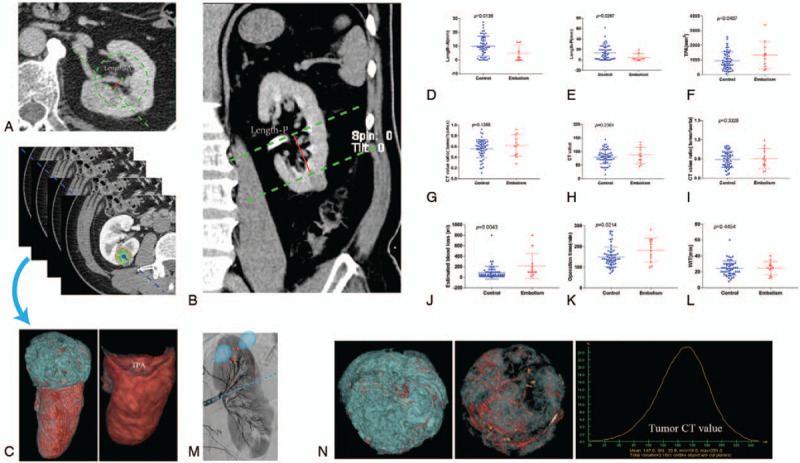
Measurement method of renal tumor-parenchyma contact area, length-A and length-P (A-C). Differences in imaging and surgical factors between embolism patients and controls (D-L). As Length-A and Length-P decreased, the order of the involved arteries increased. The red arrow shows the involved arteries (M) and measurements of the mean tumor CT value (N).

(1) Images were scrolled to the axial plane showing the largest diameter of the tumor. The kidney periphery excluding the tumor was outlined, and the center was assigned. The distance from the center point to the closest tumor edge was named Length-A and measured (Fig. 1A).^[10]^
(2) As described previously,^[10]^ image sections were counted from the upper to the lower renal borders. The renal equatorial plane was the median image section between these borders. Then, the distance from the tumor edge to the equatorial plane, called Length-P (Fig. 1B), was measured.
(3) The number of image sections from the first plane where the tumor edge was visible to the last plane was counted, and in each section, the outline of the TPA was marked and measured. The sum length of the outline multiplied by the image slice thickness yielded the TPA (Fig. 1C)

### 2.3 Embolization procedure

All patients suspected of bleeding underwent angiography in the interventional radiology department after providing informed consent. Some patients received embolization treatment after a diagnosis of pseudoaneurysm, arteriovenous fistula, or arterial bleeding was confirmed. During the operation, the patient was placed supine under local anesthesia, and a 5F vascular sheath was inserted through the femoral artery using the Seldinger technique as an introducer (Terumo, Japan). Abdominal aortography with a 5F pigtail catheter (Merit) was performed by injecting 25 mL contrast media to show the main or accessory renal arteries. Then, superselective renal angiography was performed using a 5F cobra catheter (Merit) and a 2.7F microcatheter (Terumo, Japan) to determine the hemorrhage location and pattern. The interventional radiologist placed the microcatheter as close to the bleeding site as possible and inserted the embolizing agents. All lesions were embolized with enough appropriately sized platinum microcoils (Cook) to match the offending artery. A few operations were completed by combining microcoils with gelfoam particles (Alicon, China) or polyvinyl alcohol particles (Alicon, China) for lesions that could not be reached by superselective catheterization. At the end of the operation, renal arteriography was repeated to ensure complete occlusion of the lesion and cessation of the hemorrhage. The artery puncture site was closed by Starclose (Abbott) or Perclose vascular closure devices (Abbott) and dressed with pressure bandages. The patients were asked to stay in the bed for another 6 hours to prevent hematoma formation (Fig. 4A).

### 2.4 Statistical analysis

Statistical analyses were performed using SPSS version 22.0 (SPSS Inc., Chicago, IL). Differences between the TAE and control groups were evaluated using the independent sample *t* test. Variables that showed significant differences were included in a multivariable logistic regression analysis. A model based on the multivariate logistic regression results was established to predict hemorrhage. Differences between TAE patients with hematuria and with other symptoms were evaluated using the Mann–Whitney *U* test. The mean eGFR levels were compared using independent sample *t* tests and paired *t* tests as appropriate. A *P*-value < .05 was considered statistically significant.

## 3 Results

### 3.1 Overall results

Overall, 12 (10 men, 2 women; age, 55.3 ± 12.4 years) of 810 patients (1.5%)—all of whom underwent LPN—underwent interventional radiology for postoperative hemorrhage. The 12 patients were matched to 56 patients (48 men, 8 women; age, 58.8 ± 11.0 years) without postoperative hemorrhage as a control group. Tumor size and side were assessed. The reasons for PN included clean cell renal carcinoma (Embolism vs Control, 7 vs 47) and others (Embolism vs Control, 5 vs 9). The median preoperative follow-up time was 12 months (range 0–44, Embolism) vs 16 months (range 5–46, Control). The patient characteristics are reported in Table 1. In conclusion, no significant difference between the groups was found concerning any of the patients’ characteristics.

### 3.2 Predictive factors for postoperative hemorrhage

**Figure 2:**
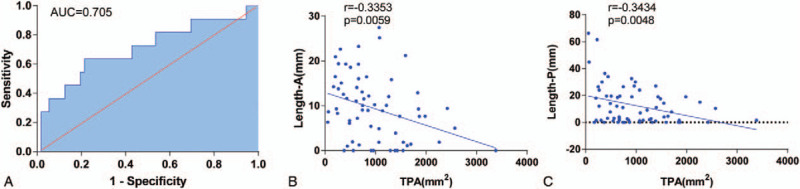
Renal tumor-parenchyma contact area and estimated blood loss discriminated embolism patients from controls, with an area under the ROC curve of 0.705 (64% sensitivity and 79% specificity) (A). The correlational analyses showed that renal tumor-parenchyma contact area was negatively associated with the value of length-A or length-P (B, C).

When compared with controls, patients with postoperative hemorrhage who underwent interventional radiology were found to have comparable mean tumor CT values (Fig. 1N), CT value ratios of tumor/aorta, CT value ratios of tumor/cortex, and warm ischemic times (*P* > .05). However, the TAE group had a significantly increased tumor-parenchyma contact area (TPA) (1329.0 mm^2^ [SD 276.0] versus 945.7 mm^2^ [SD 79.9], *P* = .041), operation time (182.3 minutes [SD 16.2] versus 149.8 minutes [SD 6.4], *P* = .021) and estimated blood loss (215.0 mL [SD 69.0] versus 88.1 mL [SD 16.1], *P* = .004). Meanwhile, Length-A (4.9 mm [SD 1.7] versus 10.1 mm [SD 1.0], *P* = .014) and Length-P (4.9 mm [SD 2.0] versus 13.2 mm [SD 1.9], *P* = .027) were significantly shorter in patients with postoperative hemorrhage than in those without (Fig. 1 D-L, Table 2). Univariable regression analyses confirmed TPA and estimated blood loss as statistically significant predictors of the occurrence of postoperative hemorrhage worthy of management with embolization, with an area under the ROC curve of 0.705 (64% sensitivity and 79% specificity) (Fig. 2A, Table 3).

**Table 2:** Patient characteristics and clinical presentations.

Parameter	Control	Embolism	*P* value
CT value (IU)	80.4 ± 3.5	86.5 ± 8.6	.238
RTA	0.49 ± 0.03	0.52 ± 0.08	.333
RTC	0.56 ± 0.03	0.62 ± 0.06	.136
TPA (mm^2^)	945.7 ± 79.9	1329.0 ± 276.0	.041
Length-A (mm)	10.1 ± 1.0	4.9 ± 1.7	.014
Length-P (mm)	13.2 ± 1.9	4.9 ± 2.0	.027
Operation time (min)	149.8 ± 6.4	182.3 ± 16.2	.021
Warm ischemic time (min)	24.4 ± 1.2	24.8 ± 2.4	.445
Estimate blood loss (mL)	88.1 ± 16.1	215.0 ± 69.0	.004

**Table 3:** Logistic regression analysis of risk factor associated with Hemorrhage.

Parameter	OR (95%CI)	*P* value
TPA	1.001 (1.000–1.002)	.048
Estimated blood loss	1.004 (1.000–1.008)	.042

**Figure 3:**
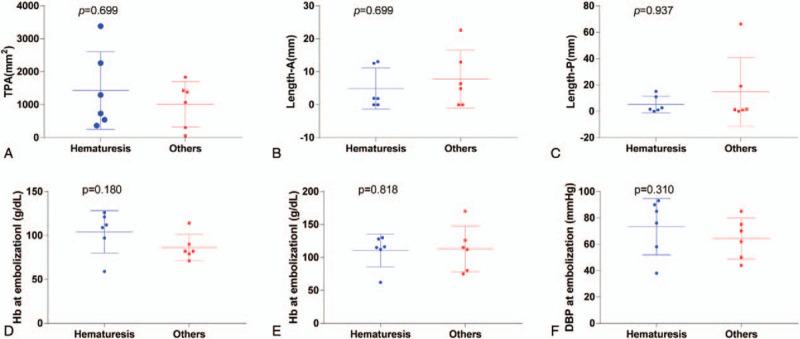
Subgroup analysis of the 12 transarterial embolization patients (hematuria vs other clinical symptoms).

In addition, we divided the 12 postoperative hemorrhage patients into 2 groups: 1 group had hemorrhage in the collection system, whose clinical manifestation was hematuria, and the other had hemorrhage in the retroperitoneal cavity with symptoms of sudden abdominal or flank pain hemodynamic or instability refractory to conservative treatment. In the subgroup analysis, no significant difference was detected between the 2 groups (Fig. 3, Table 4).

**Table 4:** Patients characteristics by Hematuria and other patients.

	Hematurian = 6	Othersn = 6	*P* value
Age (yr)	52.3 ± 11.7	58.3 ± 13.47	.485
Female n (%)	0 (0)	2 (33)	
Tumor size (mm)	3.5 ± 0.8	3.6 ± 1.1	.699
Time interval (d)	8.3 ± 9.4	4.8 ± 6.4	.466
Hb at embolizationl (g/dL)	104.0 ± 24.2	86.3 ± 14.9	.180
SBP at embolization (mm Hg)	110.5 ± 24.9	113.0 ± 34.5	.818
DBP at embolization (mm Hg)	73.3 ± 21.4	64 ± 15.5	.310

### 3.3 Clinical outcomes

**Figure 4:**
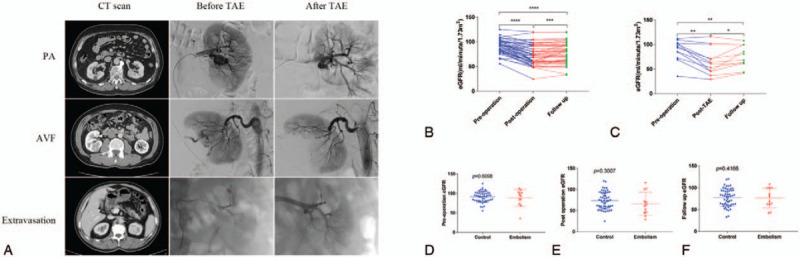
Preoperative CT scan and intraoperative findings for transarterial embolization (A). Difference in pre- and postsurgical (or transarterial embolization) and last follow-up eGFR values (B). Differences among groups at different time points (C).

Clinical success was achieved for all patients (12/12) with the following angiographic findings: PA (n = 6), AVF (n = 2), extravasation (n = 2), and no active bleeding (n = 2) (Fig. 4A). Eight middle lesions and 2 polar lesions were embolized. Nine second renal artery branches and 1 combination of second and third branches were involved. The embolic materials used for TAE included microcoils (n = 6) and a combination of microcoils and gelfoam (n = 4). Procedural details are summarized in Table 5.

**Table 5:** Details of angiographic findings and embolization techniques.

Parameter	Case number
Angiographic findings	
PA	6
AVF	2
Extravasation	2
Negtive	2
Hemorrhage position	
Middle	8
Polar	2
Arteries branches	
Grade 2	9
Grade 2 and 3	1
Embolic agents	
Microcoil	6
Microcoil and Gelfoam	4

### 3.4 Renal functions

Renal functions were assessed in all patients. The eGFRs at diagnosis of the renal mass (preoperation), after the surgery or TAE, and at the last follow-up were recorded. Statistically significant differences in renal function were observed between the different time points within groups (all *P* < .05) (Fig. 4B-C). However, no statistically significant differences were shown at the same time points (*P* > .05) between the control and embolism groups (Fig. 4 D-F). Details about the analysis of renal function are given in Table 6.

**Table 6:** Details of changes in renal functions at diagnosis of vascular complication, post-operation or transarterial embolization, and at the last follow-up.

eGFR(mL/min/1.73m^2^)	Pre-operation	post-operation (or after TAE)	At the last follow up
Control	91.1 ± 13.6	73.8 ± 20.4	78.2 ± 20.1
Embolism	88.6 ± 21.5	66.3 ± 27.5	76.3 ± 22.6

## 4 Discussion

In our patient cohort, we found a postoperative hemorrhage rate of 1.50% due to vascular complications after PN, and literature data report an incidence of vascular complications ranging from 1.96% to 5.90%.^[6,11,12]^ Vascular complications may lead to life-threatening conditions. To our knowledge, however, no study has evaluated the association between preoperative imaging parameters, as determined by contrast CT, surgical variables and postoperative hemorrhage.

We postulated that the postoperative hemorrhage resulting from vascular lesions mainly depended on

(1) tumor blood supply,
(2) the size and numbers of involved renal arterial branches, and
(3) surgical variables.

In the present study, we used the mean tumor CT value and normalized CT value (ratio of tumor/aorta and ratio of tumor/cortex) as parameters in assessing the blood supply and angiogenesis.^[13]^ Our results indicated that a more abundant tumor blood supply did not increase the risk of postoperative hemorrhage, which challenged us to be alternatively concerned about the primarily involved renal arterial branches and their numbers.^[14]^ Taking into consideration the relationship between renal tumor depth and the renal arterial vascular anatomy (Fig. 1M), as Length-A and/or Length-P decreased, the size of involved arteries also increased; thus, the risk of bleeding increased. The other key point was the number of involved arteries, which was assessed by the TPA representing the size of the surface of the wound. In our analysis, Length-A, Length-P and TPA correlated well with the risk of increased postoperative hemorrhage. However, tumor size or morphology are indirect indicators of the relationship between tumor depth and size/importance of the renal arterial branches to be addressed during PN.^[14]^ Regarding surgical variables, operation time, and estimated blood loss, used to assess the complexity of the operation, were associated with postoperative bleeding. Interestingly, univariable regression analyses confirmed TPA rather than Length-A or Length-P as a statistically significant predictor of the occurrence of hemorrhage after PN. The correlational analyses showed that TPA was negatively associated with the value of Length-A or Length-P, which suggested that the larger the TPA was, the shorter the Length-A or Length-P (Fig. 2B, C), providing an attractive single imaging index to predict postoperative hemorrhage.

According to the differences in hemorrhage sites, bleed into either the collection system or retroperitoneal cavity, the 12 patients were split into 2 groups, but the subgroup analysis did not show any significant differences in clinical characteristics. All 12 patients underwent TAE with a clinical success rate of 100%.

The renal function of both groups significantly declined post PN (or PN plus embolization) and at the last follow-up, which may have resulted from surgically induced renal damage and/or the impact of TAE^[15–19]^ (Fig. 4B, C). Due to the matched-pair approach, the 2 groups showed similar preoperative eGFR values. Furthermore, the short-term eGFR and the eGFR at last follow-up were comparable, reflecting surgically induced renal function impairment (Fig. 4D-F).

The major drawback of our study was its retrospective, single-institution nature, limiting the generalizability of the results. The small sample size and rare occurrence of postoperative hemorrhage due to artery lesions limited the statistical power of the analyses.

## 5 Conclusions

Postoperative hemorrhage is a rare but severe and potentially life-threatening complication after PN. The occurrence is associated with a larger TPA and more estimated blood loss during the procedure. Among patients who underwent selective TAE, renal function remained comparable with that of controls.

## Author contributions

**Conceptualization:** Shijun Liu.

**Data curation:** Xin Zhi.

**Formal analysis:** Shijun Liu.

**Investigation:** Caipeng Qin.

**Project administration:** Jian Gao, Tao Xu.

**Resources:** Qing Li.

**Software:** Fei Wang.

**Validation:** Qing Li, Tao Xu.

**Visualization:** Jian Gao, Tao Xu.

**Writing – original draft:** Caipeng Qin, Tao Xu.

**Writing – review & editing:** Fei Wang, Tao Xu.
